# Underweight children are agile but lack power

**DOI:** 10.1186/s12887-022-03544-3

**Published:** 2022-08-18

**Authors:** Evi Verbecque, Dané Coetzee, Bouwien Smits-Engelsman

**Affiliations:** 1 Rehabilitation Research Centre (REVAL), Rehabilitation Sciences and Physiotherapy, Hasselt University, Agoralaan Building A, 3590 Diepenbeek, Belgium; 2 Physical Activity, Sport and Recreation, Faculty Health Sciences, North-West University, Potchefstroom, South Africa; 3 Division of Physiotherapy, Department of Health & Rehabilitation Sciences, Faculty of Health Sciences, University Cape Town, Groote Schuur Hospital, Suite F-45, Old Main Building, Cape Town, 7925 South Africa

**Keywords:** Underweight, Thinness, Muscle strength, Agility, Muscular fitness

## Abstract

Given the knowledge gap in literature on the impact of undernutrition on muscular power and agility in school-aged children, the aim of this study was to compare physical fitness in such underweight- and normal weight children. In this cross-sectional study, 853 children were included (459 boys; mean age: 9.2 (1.8) years). The children were grouped according to their BMI-for-age-and-sex: normal weight (− 1 ≤ z-score < 2) and underweight (z-score < − 1). Within the underweight group, three thinness subgroups were composed: grade 1 (− 2 ≤ z-score < − 1), grade 2 (− 3 ≤ z-score < − 2) and grade 3 (z-score < − 3). Their agility, muscular endurance and power were assessed with the Performance and Fitness test battery (PERF-FIT). Regardless the country they lived in, the underweight children showed better agility (*p* = 0.012) and muscular endurance (*p* = 0.004) than those with normal weight. They presented with lower muscular power than the normal weight group, shown by significantly shorter overhead throwing distances (*p* = 0.017) and less standing long jump peak power (*p* < 0.001). The standing long jump peak power decreased further with increasing thinness grade (*p* = 0.027).

**Conclusion:** Underweight children are more agile, but have lower muscular power compared to their normal weight peers. Its relationship with motor competence and physical activity, necessitates attention for tackling muscular strength deficiencies in these children, enabling them to meet the basic requirements for a healthy lifestyle later in life.

## Introduction

Physical fitness is a powerful health marker during childhood and predicts health later in life [1–4], but is also a complex construct comprising both cardiorespiratory and musculoskeletal fitness. Cardiorespiratory fitness is the capacity of the circulatory and respiratory systems to supply oxygen to skeletal muscle mitochondria for energy production needed during physical activity and is associated with risk factors for chronic disease [5]. Musculoskeletal or muscular fitness is an umbrella term for a multidimensional construct covering the ability of a (group of) muscle(s) to exert force maximally (muscular strength), quickly (muscular power), or repeatedly (muscular endurance), but also the ability to move a joint through a full range of motion (flexibility) [6]. Muscular fitness is also related to cardiovascular risk, adiposity, skeletal health and even self-esteem in children [7, 8].

Body composition, and more specifically body mass index (BMI), a measure indicating nutritional status [9], is known to be related to physical fitness. A large body of evidence about physical fitness is available on children with overweight and obesity. For instance, compared to normal weight children, obese children have lower cardiorespiratory fitness, but better (isometric) strength [1, 2, 4]. At the other end of the nutritional spectrum, underweight is also an expression of malnutrition, but whether and how this affects physical fitness in children remains unclear. Although, the prevalence of underweight tends to decrease globally [10], it is still threefold in low- and lower-middle income countries compared to upper-middle- and high-income countries [11, 12].

The few records available on underweight and physical fitness in children, report contradictory results [1, 4, 13–16]. Consensus exists regarding cardiorespiratory fitness, which seems to be similar in underweight children and normal weight peers [1, 4, 13] or even better [14, 15]. Results on muscular fitness on the other hand are diverging: differences between children with normal weight and underweight or undernourished peers were not always in favor of the normal weight children [1, 13–16].

Due to its extensive construct, measurements for muscular fitness can vary strongly. For instance, hand grip strength (strength), overhead throw (power) or time of flexed arm hang (endurance) measure different aspects of muscular fitness [7]. Therefore, the outcome measure and its measurement unit (e.g. kg, kg/s^2^, kg/m, m, s or number of repetitions per time unit) may influence the results. Compared to normal weight peers, Bénéfice [16] found poorer throw results in undernourished Senegalese children, whereas Monyeki and colleagues [14] reported better flexed arm hang performances for undernourished South African children. Although both tasks require sufficient muscle strength, the impact of the child’s body weight plays a different role. Throwing a sand bag with a fixed weight may be more difficult for underweight children as it demands muscle strength independent from their body weight. Contrary, a flexed arm hang performance requires sufficient muscular endurance relative to the child’s body weight, making this task easier for underweighted children to perform compared to normal weight peers. Thus, body weight can influence a child’s speed, endurance, and power, whereas body composition can affect its strength, agility, and appearance [17].

In short, a knowledge gap exists in literature on the impact of undernutrition on muscular power and agility in school-aged children living in low-resourced areas. Recently a validated and reliable test for assessing motor skill related physical fitness was developed, the Performance and Fitness test battery (PERF-FIT), suited for use in low-resourced areas [18–20]. The aim of this study is therefore to investigate whether and how muscular fitness (measured by muscular power, muscular endurance and agility) in underweight school-aged children differs from that in normal weight peers.

## Methods

### Procedure

This cross-sectional study was approved by the following Human Research Ethics Committees (HREC) (North-West University HREC, NWU-00491-19-A1; University of Cape Town HREC, HREC Ref 598/2019; University of Ghana GHS-ERC, 084/04/19). Written informed consent was obtained from the parents/legal guardians before study enrolment. The children gave written assent on the test day. Data were collected between January and September 2019 by senior researchers and post graduate physical or occupational therapy students and students with a qualification in Human Movement Science, specializing in Kinderkinetics. All assessors received at least 8 hours of training.

### Participants

Twelve hundred children were invited to participate in the study and were recruited through stratified sampling as part of a larger study designed for developing and validating the PERF-FIT in low-resourced areas. The data for this study were sampled from a random group of elementary school children included in the data collection study for reference norms that took place in South Africa and Ghana. In that project, population-based sampling based on census data from 2017 was used to recruit at least 1000 children between 5 and 12 years of age from mainly from low SES background. The governmental categorization of schools and its concomitant funding was used for the selection of schools, which is based on the socioeconomic status of the community in which the schools are located. As such, the children were recruited from low-resourced areas in Ghana (four primary schools near Accra and one in the Eastern region of Ghana) and in South Africa (two schools in the Western Cape and two in North West Province). The schools where they were recruited from were located in different socio-economic areas: four schools in low socio-economic areas; three schools low-middle socio-economic areas; one school middle socio-economic areas; and one school located in a high-middle socio-economic area. The participating schools were recruited through the researchers’ network. Once the schools gave consent to participate, the children were randomly selected.

Children were included in the study if they had no signs of underlying pathologies impeding participation in physical activity such as cardiovascular (e.g. heart condition), musculoskeletal (e.g. joint or bone problems), metabolic (e.g. diabetes) or neurological (e.g. epilepsy) disorders. To check for eligibility, the parent(s) filled in the child physical activity readiness questionnaire (PARQ) [21]. Children were excluded if they had: a formal diagnosis impeding muscular fitness (PARQ), a BMI-for-age-and-sex > 25, refused testing, or incomplete test results due to absence from school during test administration.

### Measuring instruments

#### Nutritional status

The anthropometric measurements included body mass (kg), height (cm) and waist circumference (cm). Height was measured with a portable stadiometer, waist circumference with a measuring tape and body mass with an electronic scale (BF 511, Omron). Each participant’s waist-to-height ratio and BMI (kg/m^2^) was calculated. Using the BMI *z*-scores (BMI-for-age-and-sex) *Normal weight* (− 1 ≤ z-score < 2) and *Underweight* (z-score < − 1) were distinguished. Within the underweight group, three thinness subgroups were composed based on the International Obesity Task Force (IOTF) criteria: *thinness grade 1* (− 2 ≤ z-score < − 1), *thinness grade 2* (− 3 ≤ z-score < − 2) and *thinness grade 3* (z-score < − 3) [22].

#### PERF-FIT

The PERF-FIT is a reliable and valid test to assess *motor skill related physical fitness* in 5- to 12-year-old children living in low-resourced areas [18–20]. The PERF-FIT comprises two subscales: an agility and power subscale for muscular fitness and a motor skill subscale for motor competence. For this study the agility and power subscale was used, which contains five items: *running* (agility), *stepping* (agility), *side jump* (muscular endurance), the *standing long jump* (muscular power of the legs) and the *overhead throw* (muscular power of the arms) [23]. The items are described in detail in Table 1.

**Table 1:** Description of the agility and power subscale items of the PERF-FIT

Items	Description
**Running**^**a**^	The child runs as fast as possible in the ladder, one foot per square, runs around the bottle and returns the same way. The time (s) taken to complete this 8-m run and the number of mistakes (touching the ladder, stepping outside the ladder, skipping a square or losing balance) are recorded.
**Stepping**^**a**^	The child steps with two feet in each square as fast as possible, runs around the bottle and returns the same way. The time (s) taken to complete this this 8-m run and the number of mistakes (touching the ladder, stepping outside the ladder, skipping a square or losing balance) are recorded.
**Side jump**^**a**^	The child jumps sideways on its feet, with one foot per square, in the same three squares of the agility ladder. The total number of correct landings in 15 s is recorded (anaerobic muscle endurance). If toes or heels touch the sidebars of the ladder, the landing is not counted. If the child steps outside the ladder or falls on the floor, only the correct landings before losing balance are counted.
**Standing long jump**	The child jumps forward as far as possible and lands on its feet in a controlled and balanced manner. The distance between the starting line and the heel of the foot that landed closest to the starting line is measured (cm).
**Overhead throw**	The child kneels just behind the starting line and throws a sandbag (2 kg) forward as far as possible. The child holds the sandbag behind the head (starting position). The distance between the starting line and the part of the sandbag closest to the starting line is measured (cm).

*Legend*: ^a^ The running, stepping and side jump items are performed in a 3.5 m agility ladder [23]. For the running and stepping items, a bottle is placed 50 cm from the last bar of the ladder [23]

Each item is performed twice with 15 seconds in between. The best performance serves as the final result.

The outcome variables of the *running* and *stepping* items are expressed as time (seconds), for the *side jump* as number of correct jumps and for the *standing long jump* and *overhead throw* as distance (cm). Based on the jumping distance and the child’s weight and sex, the peak power of the *standing long jump* was determined:

1. Boys = (9.0*age) + (7.1* Weight (kg)) + (0.8 *Long jump (Inch)) - 97.7 [24]
2. Girls = (9.0*age) + (3.7) + (7.1* Weight (kg)) + (0.8 *Long jump (Inch)) - 97.7 [24]

### Statistical analysis

The data were analyzed using Statistical Package for the Social Sciences for Windows (Version 27.0). The sample was described using demographic data (age, sex), anthropometric data (weight, height, BMI, prevalence of stunting) and country. Normal distribution of the data was tested with the Shapiro-Wilk test. The prevalence of underweight was determined using the BMI-for-age-and-sex defined by Cole et al. [22] and expressed as a percentage of the entire sample. The groups were compared regarding sex distribution, stunting prevalence and country with a two-tailed chi-square test. Their mean age, height, weight, and BMI were compared across groups (normal versus underweight) using a one-way analysis of variance.

To determine the difference in muscular fitness based on nutritional status, Mann-Whitney-U tests were used to compare the muscular fitness performances between the normal weight children and those with underweight and in subsets defined by either country because of its significantly different distribution.

If the underweight children performed differently compared to normal weight children, the Kruskal-Wallis test as applied to identify differences in muscular fitness performance between the thinness groups. Next, the thinness groups were compared within each country because of their significantly different distributions. The level of significance was set at *p* < 0.05.

## Results

### Participants

**Fig. 1:**
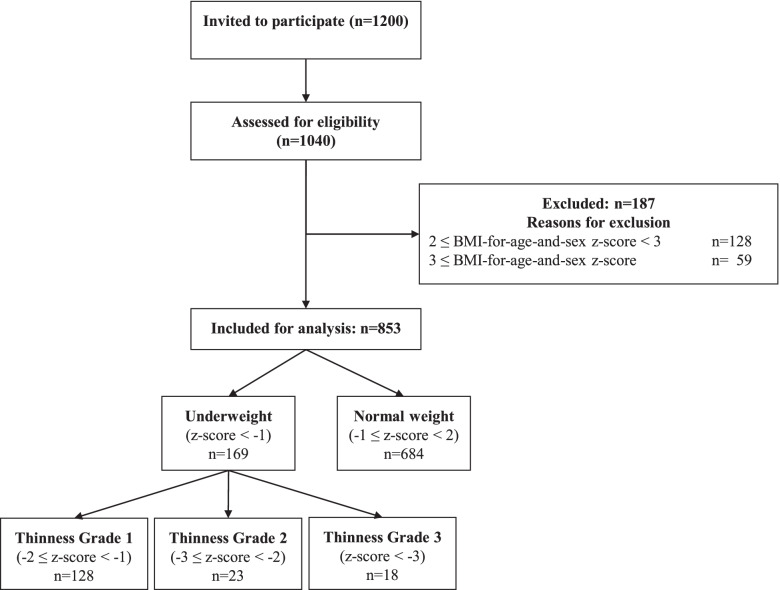
Flowchart of the selection process of eligible participants

Of the invited children, 87% (1040/1200) participated in the project, 853 of which were eligible for this study (mean (SD) age: 9.2 range 5.8–12.9 years; mean (SD) height: 133.9 (11.8) cm; mean weight (SD): 28.71 (7.17) kg; 459 boys/394 girls). Figure 1 depicts the selection process of included children. In total, 19.8% of the children were underweight.

The normal- and underweight group had an equal distribution of boys and girls (*X*^*2*^ = 0.278, *p* = 0.598), number of stunted children (*X*^*2*^ = 0.04, *p* = 0.948) and a similar mean height (F_1,851_ = 0.532, *p* = 0.466). The underweight children were significantly older (F_1,851_ = 4.367, *p* = 0.037) and significantly fewer of them went to schools situated in high socio-economic areas. Both groups are described in Table 2.

**Table 2:** Description of the Normal weight and Underweight groups

	Groups		***p***-value
	Normal weight	Underweight	
Boys/girls (N)	365/319	94/75	0.598^a^
Country: Ghana/South Africa (N)	327/357	121/48	**< 0.001**^**a**^
Age (years, mean (SD); range)	9.2 (1.8); 5.9–12.9	9.5 (1.7); 5.8–12.9	**0.037**^b^
Height (cm, mean (SD); range)	133.8 (11.9); 107–167	134.5 (11.6); 111–185	0.466^b^
Weight (kg, mean (SD); range)	29.69 (7.25); 16.2–56.7	24.75 (5.24); 11.6–41.1	**< 0.001**^b^
BMI (kg/m^2^, mean (SD); range)	16.32 (1.51); 13.89–21.39	13.55 (1.27); 6.00–15.21	**< 0.001**^b^
Waist-to-height ratio (mean (SD); range)	0.445 (0.036); 0.32–0.60	0.418 (0.030); 0.36–0.50	**< 0.001**^b^
Stunting (N)	25	6	0.948^a^
Socio-economic area			
Low	288	81	**0.039**^**a**^
Low-middle	281	73	
Middle	52	10	
High	**63**	**5**	

*Legend*: ^a^ two-tailed Chi-square test; ^b^ one-way analysis of variance. Bold values indicate significant differences

Within the underweight group, the *thinness grade 1* group consisted of 128 children (71 boys), the *thinness grade 2* group of 23 children (15 boys) and the *thinness grade 3* group of 18 children (8 boys). The thinness groups had a similar sex distribution (*X*^*2*^ = 1.77, *p* = 0.413), mean age (F_2,166_ = 0.349, *p* = 0.706), mean height (F_2,166_ = 1.326, *p* = 0.268) and went to schools located in similar socio-economic areas. Weight (F_2,166_ = 6.962, *p* = 0.001) differed significantly between the groups (*thinness grade 1* = *thinness grade 2* < *thinness grade 3*). As defined by the group composition, BMI was significantly different between the three thinness groups (F_2,166_ = 150.428, *p* < 0.001). Table 3 provides a detailed description of the three groups.

**Table 3:** Description of the Underweight subgroups

	Groups			***p***-value
	Thinness 1	Thinness 2	Thinness 3	
Boys/girls (N)	71/57	15/8	8/10	0.413^a^
Country: Ghana/South Africa (N)	87/41	18/5	16/2	**< 0.001**^**a**^
Age (years, mean (SD); range)	9.5 (1.7); 5.8–12.9	9.6 (1.8); 6.0–11.9	9.2 (1.6); 6.0–11.8	0.706^b^
Height (cm, mean (SD); range)	133.7 (10.9); 111–161	137.1 (12.3); 113–163	137.1 (15.5); 119–185	0.268^b^
Weight (kg, mean (SD); range)	25.33 (4.85); 16.8–38.7	24.80 (4.60); 16.2–34.6	20.57 (6.92); 11.6–41.1	**0.001**^b^ 1 = 2 > 3
Waist-to-height ratio (mean (SD); range)	0.422 (0.028); 0.37–0.50	0.404 (0.028); 0.36–0.45	0.409 (0.035); 0.36–0.47	**0.014**^b^ 1 > 2 = 3
BMI (kg/m^2^, mean (SD); range)	14.03 (0.54); 13.01–15.21	13.08 (0.36); 12.47–13.81	10.74 (1.82); 6.00–12.48	**< 0.001**^b^ 1 > 2 > 3
Socio-economic area				
Low	56	13	12	0.405^a^
Low-middle	59	8	6	
Middle	8	2	0	
High	5	0	0	

*Legend*: ^a^ two-tailed Chi-square test; ^b^ one-way analysis of variance. Bold values indicate significant differences

### Muscular fitness

#### Performance of normal weight versus underweight children

As shown in Table 4, the underweight children showed better agility and endurance compared to those with normal weight. During the stepping item they needed less time to complete the task successfully (*p* = 0.012) and they performed more side jumps during the 15-second time window (*p* = 0.004). They presented with lower muscular power than the normal weight group, shown by shorter overhead throwing distances (*p* = 0.017) and less standing long jump peak power (*p* < 0.001). Both groups performed similar for running (*p* = 0.492) and the standing long jump distance (*p* = 0.155).

**Table 4:** Muscular fitness performance in groups classified according to BMI-for-age-and-sex status

	Normal weight	Underweight	***p***-value*
	Median (IQR)	Median (IQR)	
Running (s)	7.52 (1.96)	7.42 (1.73)	0.492
Stepping (s)	15.65 (5.33)	14.79 (4.34)	**0.012**
Side jump (number of jumps)	22 (12)	25 (12)	**0.004**
Standing long jump (cm)	116.0 (35.0)	119.0 (31.3)	0.155
Standing Long jump peak power (Watt)	223.68 (101.01)	197.97 (78.66)	**< 0.001**
Overhead throw (cm)	215.5 (84.0)	198.0 (69.0)	**0.017**

*Legend*: * *p*-values are extracted from Mann-Whitney-U tests; bold values indicate significant differences

Though more children belonged to the thinness groups in Ghana, the same results were seen in their muscular fitness performances. Normal weight children performed similar to underweight peers in both countries on all tasks, except for the overhead throw (*p* < 0.05) and for the standing long jump peak power (*p* < 0.001).

#### Performance of underweight children: comparing thinness grades

Overall, muscular fitness measures were similar across the thinness groups (Table 5), except for the standing long jump peak power (*p* = 0.027).

**Table 5:** Muscular fitness performance in thinness groups

	Degrees of underweight			
	Thinness grade 1 (***n*** = 128)	Thinness grade 2 (***n*** = 23)	Thinness grade 3 (***n*** = 18)	***p***-value
	Median (IQR)	Median (IQR)	Median (IQR)	
Stepping (s)	14.98 (4.61)	15.93 (4.77)	13.96 (2.70)	0.194
Side jump (number of jumps)	25 (12)	21 (16)	26 (9)	0.897
Standing Long jump peak power (Watt)	200.05 (73.66)	211.14 (84.71)	158.94 (95.72)	**0.027** **1 = 2 > 3**
Overhead throw (cm)	200.0 (70.5)	197.0 (73.0)	185.5 (45.8)	0.674

*Legend*: *p*-values are extracted from Mann-Whitney-U tests; bold values indicate significant differences

**Fig. 2:**
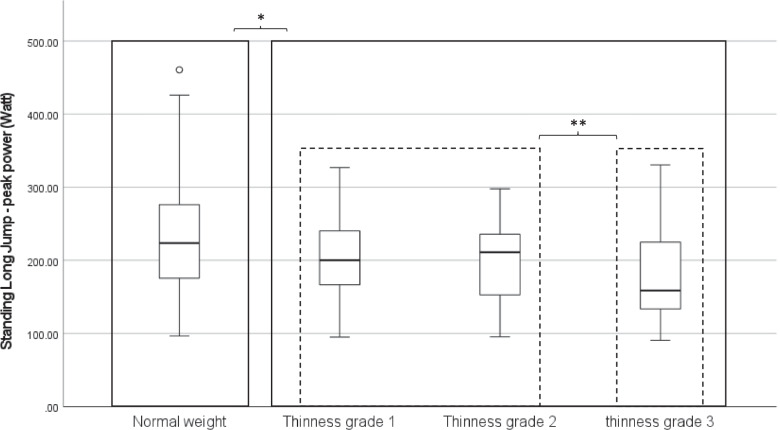
Comparison of the Standing Long Jump peak power among weight groups

As shown in Fig. 2, children in the *thinness grade 3* group had significantly lower results compared to those with *thinness grades 1 and 2 groups* (*p* < 0.05), but no differences were found between *thinness grade 1 and 2* (*p* = 0.757). Though more Ghanaian children belonged to the thinness grade 2 and 3 groups, their muscular fitness was comparable, except for their standing long jump peak power. In the Ghanaian sample, the children in thinness grade 3 had significantly lower standing long jump peak power than the other underweight children, whereas no such differences were found in the South African sample.

## Discussion

The aim of this study was to investigate whether and how muscular fitness in underweight school-aged children differs from that of normal weight peers. Overall, underweight children outperformed normal weight children on agility and muscular endurance tasks, but performed significantly poorer on muscular power tasks. Especially, the standing long jump peak power was lower in all underweight children. Thus, the type of muscular fitness task seems to play a significant role.

Underweight children are often hypothesized to have greater muscle strength relative to their weight [2]. Our results reject this hypothesis. The standing long jump peak power, which corrects the jump distance for weight, showed significantly lower power in underweight children compared to their normal weight peers, that decreased even further with increasing thinness. Similar to our findings, previous studies also showed that the overhead throw is significantly poorer in underweight children [1, 25]. These results indicate that these children do not have greater muscle strength relatively to their weight but that they have difficulties generating muscular power.

Scientific records indeed indicate that undernutrition adversely impacts muscle mass, which is associated with functional deficits [26]. Previous research has shown that lean subjects have more type I and less type IIb muscle fibers [27, 28]. Although we did not measure lean mass, but estimated nutritional status with an anthropometric proxy for body composition, the underweight children in our sample did present with functional deficits. Therefore, the muscle type fibers may explain the functional deficits in our underweight children. Where type I muscle fibers are slow-twitch fibers, that operate on the aerobic metabolism (slow but resistant to fatigue), type IIb fibers are fast-twitch operating on anaerobic capacity (fast but easily fatigued) [28]. Loss of muscle mass is common in malnourished children [29], and seems to be characterized by “a decrease in size and number of fast-twitch fibers, whereas the slow-twitch-fibers are spared” [1]. Tasks requiring muscular power may therefore be difficult for underweight children. Agility requires great maneuverability and speed, but also muscle endurance. Our underweight children outperformed normal weight peers on agility tasks. Moving less mass requires less energy, which is biomechanically more efficient, and can therefore explain why underweight children show better agility than their normal weight peers. Thus, being underweight seems to be a relative advantage at least for agility skills.

The distribution of underweight differed between countries as can be expected based on their wealth and economy (Ghana: lower middle-income country; South Africa: upper middle-income country). Nevertheless, the same differences between the groups were observed, illustrating that the adverse effects of underweight transcend national borders. Although the children’s raw physical fitness scores may differ between countries, indicating attention is needed for specific reference values in different geographical areas, the impact of being underweight seems to be universal. Insights into how undernutrition affects different aspects of muscular fitness may enable the development of clinical and public health interventions (e.g. physical education at school and leisure activities) that are more effective in promoting long-term quality of life, through reversing muscle deficits and their functional consequences.

### Study limitations

Stunting is an often-used proxy for children’s broader developmental status [13, 30], which was only present in a small portion of our children and equally spread across the normal- and underweight groups. Our sample therefore seems to differ significantly from others [1, 13, 15].

We used BMI to estimate the children’s nutritional status in terms of normal weight or underweight, but did not record their daily nutritional intake. In South Africa, the National School Nutrition Program provides one nutritious meal to all learners in poorer primary and secondary schools (quintile 1, 2 and 3 schools) (https://www.gov.za/faq/education/what-national-school-nutrition-programme-nsnp), consisting of protein (Soya, Fish, Eggs, Milk, Sour milk, Beans and Lentils), fresh fruit and vegetable, carbohydrate/starch. Especially proteins are paramount for optimal muscle mass development [31]. The WHO recommends a protein intake of 0.75–1.12 g/kg/day between 6 months and 10 years for healthy children and even more for malnourished children [32]. By mapping their calorie intake combined with skinfolds and/or circumference measures, such as the middle upper arm circumference, real undernutrition could be distinguished from naturally lean physique in future studies.

Children living in disadvantaged circumstances have fewer chances of participating in organized physical activity and sports [33–35]. Due to the interrelatedness, the children’s actual physical activity and sedentary status could have provided insights into their physical fitness and should be mapped in future research.

### Recommendations for future research and clinical practice

When investigating the impact of malnutrition on muscular fitness, the applied classification tends to influence the results and should therefore be considered. Some authors refer to malnourished children by combining a group of stunted, wasted and/or underweight children [14, 16], whereas others compare these specific subgroups [1, 2, 4, 13, 15]. Distinguishing underweight, stunted (height-for-age < − 2 z-score [36]) and wasted (weight-for-height < − 2 z-score [36]) children when comparing them to normal weight peers improves the sensitivity of performance measures to detect muscular fitness deficits [1, 13, 15]. To disentangle the impact of malnutrition on muscular fitness, subclassification of nutritional status seems needed.

Scientific records indicate that fat mass is relatively preserved over time at the expense of fat-free mass (i.e. muscle mass) because it provides energy and molecular substrates for immune function [26] when nutritional intake is depleted. Future research should therefore tackle the question whether the muscle power deficits, as seen in our results, are a consequence of muscle loss, i.e. sarcopenia [31]. This requires measures of muscle mass and fiber type in normal weight and underweight children. Furthermore, the interaction between nutritional intake, especially proteins, and lifestyle factors are determinants for body composition and muscle development [31] and should be tracked in future research.

The increasing number of children surviving malnutrition, underpins the need for a better understanding of its long-term impact [26]. Although being underweight at a young age might not have a major impact on muscular fitness yet, it could lead to more consistent impairments at an older age if not corrected for [25]. With increasing age and onset of puberty, combined with unhealthy food patterns, the children being underweight may become overweight or even obese if physical activity is not being promoted and a sedentary lifestyle is maintained. As muscular fitness phenotypes track from childhood to young adulthood [37], stimulating physical activity early-on is imperative. If muscular strength deficiencies are present in children and not corrected for, they may develop difficulties in daily moderate-to-vigorous physical activities that are required for overall participation [38], thereby developing a vicious cycle of inadequate muscle strength – inactivity and the subsequent health consequences. Thus, promoting optimal muscular fitness development as early as possible is extremely important [25].

## Conclusion

Underweight children are more agile, but have poorer muscular power compared to their normal weight peers. Due to its consistent relationship with motor incompetence, physical inactivity, and lower participation, tackling muscular strength deficiencies in these children is imperative.

## Data Availability

The datasets used during the current study are available from the corresponding author on request.
